# Benefits of using virtual reality in cariology teaching

**DOI:** 10.1186/s12909-024-05980-4

**Published:** 2024-09-27

**Authors:** Hamdi Hamama, Ka Yan Harrison, Sukhdeep Murbay

**Affiliations:** 1 Conservative Dentistry Department, Faculty of Dentistry, Mansoura University, Mansoura, Egypt; 2 Faculty of Dentistry, The University of Hong Kong, Hong Kong, China; 3 Department of Psychology, MD college of Health, University of West Florida, Pensacola, USA; 4 University of Manchester, Manchester, UK

**Keywords:** Cariology, Dental education, Haptic, Simulation training, Virtual reality

## Abstract

**Background:**

Virtual Reality (VR) has been widely used as an useful educational tool in modern dentistry and is considered as an alternative training tool adjunct to conventional training methods.

**Objective:**

This study was designed to investigate the effectiveness of VR haptic-enhanced simulators for training undergraduate dental students during practical cariology pre-clinical caries excavation sessions.

**Methods:**

A total number of 76 students were recruited for this study. Students were randomly divided into 2 groups (38 students each). The experimental group (VR-Start group), students performed caries removal at the VR haptic-enhanced simulator prior to practicing on natural extracted teeth. Conversely, the control group (Natural Tooth-Start Group), students exposed to VR simulation training after practicing on extracted natural teeth. An evaluation questionnaire was disseminated among students to evaluate their self-confidence, perceived clinical skills and their perception of providing better care to patients in the future. They were also invited to express their opinions on the usefulness of VR simulator in comparison with conventional learning methods.

**Results:**

The outcome of Chi-square test showed no significant difference in students’ response among this study groups (*p* > 0.05). Moreover, the outcome of this study revealed that both student groups considered virtual reality as a useful learning tool. Majority of students (90%) superiorly ranked experience gained from practicing on natural carious teeth. They also clearly stated that virtual reality allowed them to practice more and improve their self-confidence level as well as eye-hand coordination.

**Conclusions:**

Virtual reality simulator is a useful learning tool which can benefit undergraduate dental students at their pre-clinical stage; but, it cannot totally replace the conventional caries excavation.

## Introduction

Virtual Reality Learning Environment (VRLE) was firstly introduced in the aviation, automobile and military training. Recently, the use of VRLE with advanced technology has become more prominent in the medical training [1]. VRLEs simulate the real world through application of three dimensional (3D) models that initiate interaction and immersion of the learner. The interactive environment reinforces the sensation of the learner to the computerized virtual world. The VRLE also simulates a realistic and safe environment for learners to perform specific and complicated tasks [2].

Simulation in health care profession has been employed since 1960s when mannequins was introduced in the anesthetic training [3]. In 1990s, first simple laparoscopic simulators were developed due to the expanding interest of minimally invasive surgery [4]. Evidence shown that psychomotor skills gained in the VRLE could be transferred into real life application [5, 6]. 

In the field of dentistry, simulation of true clinical conditions is very challenging. The clinical competence of a dentist requires the assimilation of large amount of biomedical sciences and clinical knowledge together with acquisition of clinical skills and problem-solving ability. Clinical skills include physical examination and patient consultation, as well as performing clinical procedure which psychomotor skills are involved. Dentistry has extensively use simulators for training purposes, especially in the preclinical setting [7]. These simulators act as a gateway to ensure smooth transition to clinics [8]. Also, they can support and reinforce ergonomics and broaden students’ preclinical experience with additional models mimicking real patient conditions and offer practice in less-stressful environment [1].

Natural teeth were used before the introduction of the phantom head in the early days of dental training. With the innovative and advanced technology incorporated, students are now trained under virtual reality clinical simulators. Haptic enhanced simulator has been a major breakthrough in medical and dental training [9]. Haptic simulator improves the realism of the simulation and enhance the immersion experience of the users. VRLE with haptic feedback is increasingly used in medical care training, especially in the field of dentistry to allows students to develop their required psychomotor skills and techniques and qualified dentists to update their current practice safely through continue education for professional development [10]. 

With the advanced technical capabilities of VR, the system provides the sensory stimuli (visual, auditory, smell and haptic feedback) which draws the users’ subjective psychological and physiological response to the environment. The internal psychological processes of users in a VR world determine the perceived information of the users – what they see, hear and feel thus influence the learning outcomes of an individual [11]. Use of haptic feedback has appreciated as a niche foothold for replicate surgical skills training. Review from Van der Meijden et al. [12] found that the haptic feedback simulator helped reducing surgical error and important in the early phrase of psychomotor skill acquisition and development. Sewel*l et al.* [13] suggested haptic training allows students acquire the skills along the learning curve and bring the skills into reality.

Motivation, the psychological factor, has found being a potentially important factor affecting the learning effectiveness. Users motivation influences their performance including attention, effort, quality, behavior, score and grades [14]. Studies in educational psychology indicated that intrinsic motivation and academic achievement are positively correlated [15]. One of the main advantages of using VR is allowing control and active learning. These terminologies refer to the psychological state of the learner, taking the control on his/her learning. Through VRLE, learners make their own decisions on the learning content, pace, sequencing and frequency, content of instruction and amount of specific practice in the learning environment. Kinzie et al. [16] suggested that some degrees of learners’ control are important in the learning process. Learners actively involved and have the control in their learning are more competent, self-determine, intrinsically more motivated in learning [17]. Furthermore, the interactive dynamic learning environment provides the flexibility to meet learners own cognitive needs and their skills and knowledge acquired can be better comprehend and assimilated. Thus, it advances the memorization, understanding, application of the skills and knowledge.

Majority of studies showed that VR can enhance performance [7, 18–20]. Nonetheless, there is a significant lack of evaluation on how VR related to the confidence and consequently the overall enhancement of perceived clinical skills. In medical and health profession educational studies, a theoretical framework is highly demanded. Therefore, this study was conducted on bases of self-determination theory. This theory seeks to explain how being self-determined impacts motivation that people feel more motivated to act when they think that what they do will have an effect on the outcome. This allows participating dental students to know about how this theory works, as well possible ways to improve their self-determination skills. This study was designed to investigate the effectiveness of VR haptic-enhanced simulators for training undergraduate dental students during practical cariology pre-clinical caries excavation sessions. The null hypothesis is that there is no significant difference in the enhancement of perceived self-efficacy (confidence) and perceived learning outcome (clinical skills) between the VR-Start group and Natural-Tooth-Start group.

## Materials and methods

### Study design

Ethical approval of the study was granted by the University of Hong Kong ethics research board. Clinical Research Ethics- IRB Institutional Review Board of University of Kong Ref Number UW-17-034; and written informed consent was obtained from all participants. The present study was underpinned by the self-determination theory, proposed by Deci and Ryan [21]. The theory highlights that virtual reality will improve caries excavation practice. In the present content, the authors hypothesized that dental students will be motivated in their learning via VR which may enhancement their perceived self-efficacy (confidence) and perceived learning outcome.

**Fig. 1:**
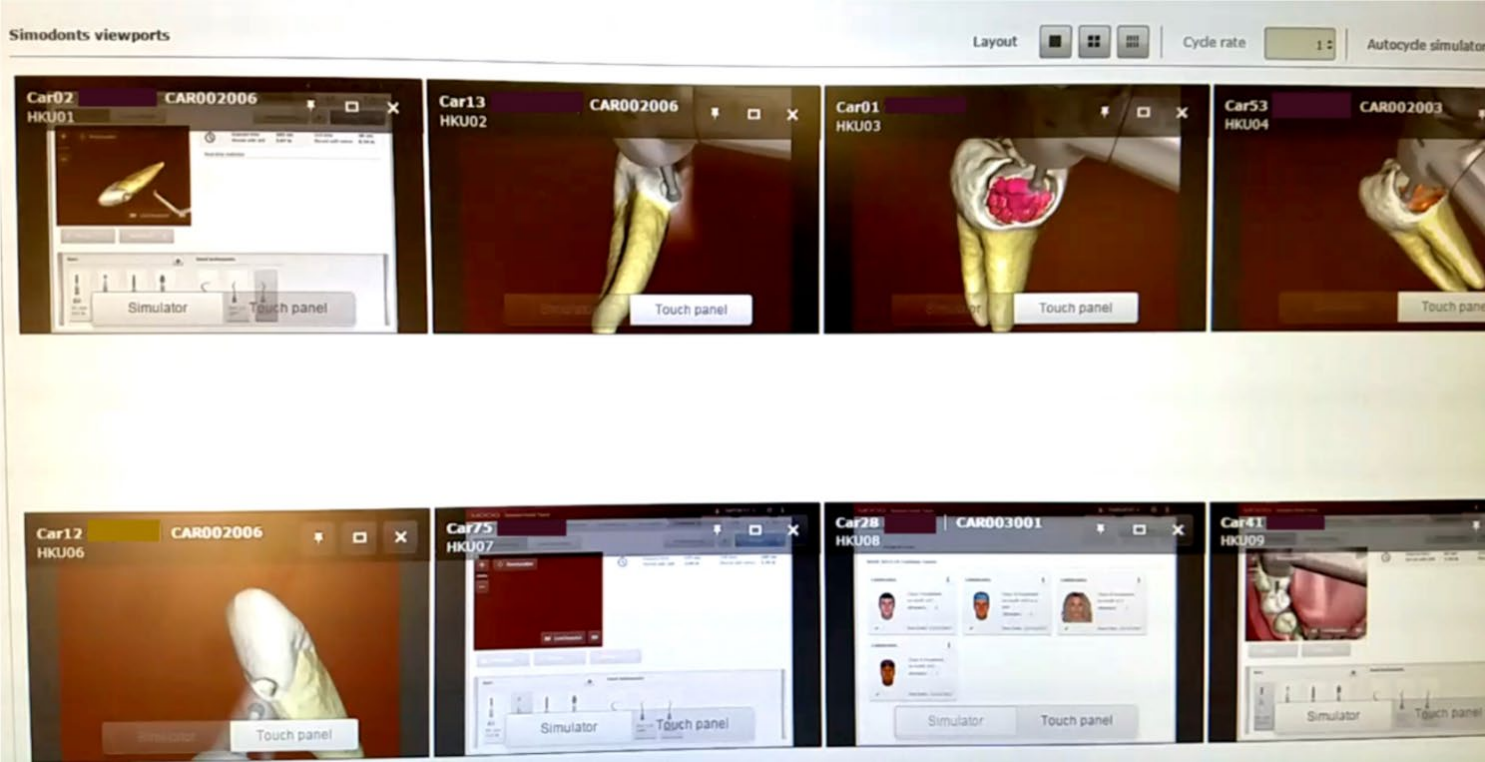
Live screen from the master control server on haptics VR simulators showing different tasks including the application of caries detector dyes and virtual clinics

A total number of 76 pre-clinical second-year dental students from the University of Hong Kong voluntarily participated in this study. They were randomly divided into 2 groups (38 students each); VR-Start group (experimental group) and Natural Tooth-Start group (control group) using an auto generated computerized method. In VR-Start group, students performed caries removal at the VR haptic-enhanced simulator prior to practicing on natural tooth. Conversely, students in the control group practiced on the extracted natural tooth prior to exposure to VR simulator experience. A questionnaire was disseminated among students, prior to the caries excavation cariology practical session, to evaluate their perceived self-efficacy (confidence); perceived learning outcome (clinical skills), perception of providing better care to patients in future. They were also invited to express their views regarding the overall learning experience of using VR simulator through an open-ended questionnaire (Fig. 1). The questionnaire sheets were collected after the students practical-session training on both natural tooth and the haptic enhanced simulator.

### Apparatus and questionnaire

The simulator was invented by the Academic Centre for Dentistry Amsterdam (ACTA). This haptic enhanced VR simulator (Cariology software, Simodont Dental Trainer, Moog B.V, Nieuw-Vennep, Netherlands) provides a high-fidelity force feedback on the basis of the unique admittance control paradigm of the Haptic Master. A pre-sit standardized caries excavation task was assigned to all students by the software administrator. Each student accessed the haptic software with his/her student ID and unique sent to his email. All participating students received a 10-mins introduction talk about instruction of using haptics at beginning of each session by one instructor. Furthermore, all the steps and instructions were clearly written in the VR simulation laboratory board.

**Fig. 2:**
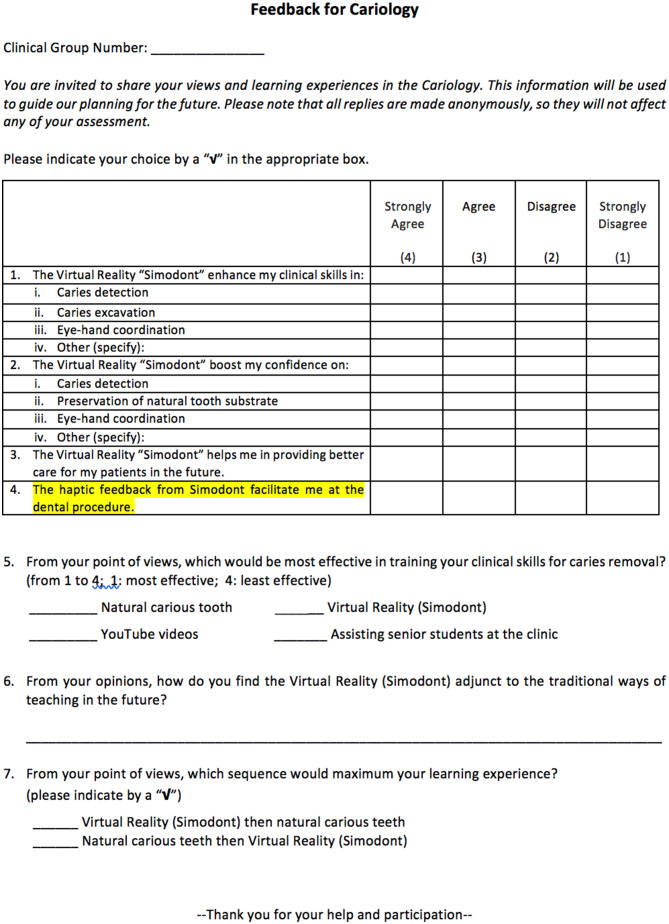
The questionnaire used in this study (The questions were designed by applied psychologist (Ka Yan Harrison)

A 4-point Likert scale (4: Strongly Agree; 3: Agree; 2: Disagree; 1: Strongly Disagree) questionnaire was developed to evaluate students’ perceived learning outcome (clinical skills), perceived self-efficacy (confidence) in varieties of items, their perception on providing better care for patients in future with the aid of VRLE and the facilitation of haptic feedback simulator for dental procedure. In the questionnaire, we also asked students to indicate their preference provided with four options: (i) natural carious tooth, (ii) virtual reality, (iii) YouTube videos, and (iv) assisting senior students at clinic to evaluate with respect to their views on effective clinical skills training in caries removal. They were also asked to express their opinions on how the virtual reality could be adjunct to the conventional teaching (Fig. 2).

### Variables and data analysis

Statistical analyses were performed between the VR-Start group and Natural Tooth-Start groups using Chi-Square test to determine whether there is a significant relationship of the perceived self-efficacy (boost in confidence), perceived learning outcome (enhancement in clinical skills), providing better care for patients in the future and the haptic feedback for dental procedure facilitation with related to the haptic enhanced simulator training. The influence of the use of haptic enhanced simulator is the independent variable (IV); while the perceived self-efficacy (boost in confidence), perceived learning outcome (enhancement in clinical skills), providing better care for patients in the future and haptic feedback for dental procedure facilitation are the dependent variables (DV). The level of significant was set at a p value less than 0.05. The data were statistically analyzed using the SPSS Software (V.23, IBM, NY, USA).

## Results

Out of the 76 questionnaires administered, a total of 67 valid returns were included in the analysis, with a response rate for the entire questionnaire questions 88.16%. Of those 67 returned questionnaires, 31 were collected from the VR-Start group; 36 were collected from the Natural Tooth-Start group. Students, not confining to VR training group or conventional training group, majority of them expressed strongly agreement or agreement that the VR haptic enhanced simulator would enhance their perceived learning outcome (i.e. clinical skills), in terms of caries detection, caries excavation and eye-hand coordination (Table 1).

**Table 1:** Overall students’ perception for the enhancement of clinical skills

Enhanced clinical skills in:	Strongly Agree	(%)	Agree	(%)	Disagree	(%)	Strongly Disagree	(%)
Caries detection	5	7.5	52	77.6	10	14.9	0	0
Caries excavation	13	19.4	47	70.2	7	10.4	0	0
Eye-hand coordination	18	26.9	40	59.7	7	10.4	2	3

Majority of students strongly agreed and agreed that VR haptic enhanced simulator would enhance their perceived learning outcome (i.e. clinical skills), in terms of caries detection, caries excavation and eye-hand coordination, there were no significant evidence to suggest an association between the perceived enhancement of clinical skills to the way of learning. There was no association found between the enhancement of clinical skills in terms of caries detection, caries excavation and eye-hand coordination and the way of learning Χ2(2) = 1.512, *p* = 0.469, Χ2(2) = 0.381, *p* = 0.827 and Χ2(2) = 0.995, *p* = 0.802 respectively.

In terms of the perceived self-efficacy (boost in confidence), students, not confining to VR training group or conventional training group, they were in strongly agreement and agreement that the haptic enhanced simulator would boost their perceived self-efficacy, in terms of caries detection, preservation of natural tooth substrate and eye-hand coordination (Table 2).

**Table 2:** Overall students’ perception for the boost of perceived self-efficacy (confidence)

Boost confidence in:	Strongly Agree	(%)	Agree	(%)	Disagree	(%)	Strongly Disagree	(%)
Caries detection	5	7.5	49	73.1	14	20.9	0	0
Preservation of tooth substrate	11	16.4	40	59.7	14	20.9	2	3
Eye-hand coordination	16	23.9	40	59.7	9	13.4	2	3

The results showed that there was no association between the boost of perceived self-efficacy (confidence) and the way of their learning in terms of caries detection, preservation of natural tooth substrate and eye-hand coordination Χ2(2) = 2.231, *p* = 0.328, Χ2(2) = 0865, *p* = 0.834 and Χ2(2) = 1.284, *p* = 0.733 respectively. With regards to students’ perception on having better patient care in the future, 84.6% of them strongly agreed or agreed the VR simulator would help them providing better care for their patients in the future. Their perception of providing better care for their patients was independent of their learning method for caries removal Χ2(2) = 2.132, *p* = 0.344.

Nearly 90% of students expressed that they strongly agreed (16.4%) and agreed (73.13%) the haptic feedback at the simulator facilitated them for the dental procedure. Nevertheless, we found the learning method, whether learnt from a VR simulator or by natural tooth, was independent of students’ perception on the haptic feedback facilitating their learning on dental procedure Χ2(2) = 0.303, *p* = 0.860.We had provided four options: (1) Natural carious tooth, (2) Virtual Reality, (3) YouTube video and (4) Assisting senior students at the clinic; for students to indicate and rank their views on the effectiveness of training their clinical skills for caries removal, with the indication of 1: most effective; 4: least effective. A total of 58 (out of 67) valid answers of this survey questions; out of these 58 valid questionnaires, 57 had indicated that they rated natural carious tooth as the most effective training tool compared with only 1 rated virtual reality simulator as most effective. In Natural Tooth-Start group; all the students rated natural tooth as the most effective learning tool for training clinical skills in caries removal. Interestingly, students rated virtual reality effectiveness (rated 2 and 3) extended 27 and 23 counts respectively. Thirty-five (out of 57) students rated assisting senior students at clinic as the least effective (rated 4).

More than half of students (58.2%) had expressed that practicing through virtual reality haptic enhanced simulator prior to working on natural tooth would maximize their learning experience. While 41.8% students commented that working on natural tooth then proceeded to virtual reality haptic enhanced simulator would maximize their learning experience. Interesting, within the group students preferred practicing through virtual reality simulator prior to working on natural tooth, 46.2% were from the VR-Start group. In other words, 53.8% of students expressed preferring VR simulator training prior to natural tooth practice for maximizing their learning experience were from the Natural Tooth-Start group. Also, they clearly stated that virtual reality allowed them to practice more and improve their self-confidence level as well as eye-hand coordination.

The student responses to the questionnaire’s open ended-questions showed that most of the students enjoyed the virtual reality training environment. The majority of comments were positive; e.g. “The learning experience is interesting and unique; fun which makes learning very engaging with the use of technology.”; “I think it gives junior students some practical and basic idea before practical courses, which is good.”; “More practicing and can work without pressure on Simodont for better learning experience.”; “Help to learn more about carious teeth”.

## Discussion

Virtual reality with haptic enhanced simulation training in dental education has been a relatively new adjunction to conventional teaching. The outcome of this study showed that the majority of students agreed that the VR haptic-enhanced simulator improved their clinical skills and increased their confidence level. They were also satisfied by the experience gained from their training on natural tooth (conventional method). This might explain the non-significance differences among VR-Start and Natural Tooth-Start groups in terms of perceived self-efficacy and learning outcomes. This was the first exposure of VR haptic-enhanced simulators for the students.

The questionnaire results revealed that students enjoyed the features of VRLE which helps to facilitate the educational process in terms of hand-eye coordination in a comfortable educational environment. They had expressed “Good for hand eye coordination”; “Improves students’ hand skill”; “Can do practice anytime during office hours”; “We can repeat the procedure as many times as we want”. These findings were in support of the study by Dutã et al. [22] who concluded that the availability of virtual reality training could provide flexibility to students for learning. Consequently, approximately 80% of students strongly agreed/ agreed the virtual reality simulator could bring positive impact towards their perceived self-efficacy (increase in confidence level) and learning outcome (clinical skills). This also can be attributed to the VR unique learning experience and convenience. The positive feedbacks from students were in accordance with the findings from Gottlieb [23] that virtual reality simulation training would allow students feeling more prepared for clinical practice.

Leopold [24] reported that VR simulation training at surgical tasks significantly help surgeons to achieve better operational confidence. Consequently, with further developments of psychomotor skills. These findings were also in total agreement with the outcome of the study by Gottlieb [25], they reported that students with VR simulation training performed better in their psychomotor skills and ability to prepare teeth for simple operative procedures than students without VR simulation training. Furthermore, the outcome of this study was in agreement with Daud et al. [20] study who reported that novice dental students generally perceived VRHS as a useful tool for enhancing their manual dexterity. Dental institutions should endorse virtual reality technology with caution, ensuring a planned integration into the curriculum to optimize benefit. Moreover, the outcome of this study agreed with Alvitez-Temoche et al. [26] who stated that VR can provide dental students with immersive, hands-on learning experiences, which can enhance their understanding and clinical skills. Furthermore, the translational value of this study extends beyond dental education. In addition the current study output are in full agreement with Plessas [27] study which revealed that the virtual augmented educational methods cannot be used as the sole training method, and the facilitator’s input is still critical.

It is believed that the acquisition of psychomotor skills and smooth coordination of gross and fine voluntary movement would enhance the clinical performance, such as tooth substrate preservation. Urbankova [28] concurred with the fact that the augmented features of virtual reality simulator could facilitate better eye-hand coordination and reduce the procedural errors; consequently, it would potentially enhance the learning efficiency and skills development. The VR haptic enhanced simulator provided a platform for novice and experienced learners to practice at their own pace and training content. For those who are low at eye-hand coordination, visual-spatial ability could benefit from the convenience of training provided by the VR haptic enhanced simulator to improve their performance and increase their confidence in clinical practice.

Regrettably, the VR haptic-enhanced simulator could not provide the representational fidelity and force feedback/ tactile sensation as students expected. Students had commented “The texture of teeth in Simodont are quite different from the natural teeth”; “The Simodont doesn’t give the same feedback (texture) than I experience while using natural teeth. The hand piece is too powerful or the teeth is too soft.”; “The texture can be improved, like the burs are too easy to remove, not like the reality”. The failure of the haptic feature might deteriorate students’ impression and satisfaction of using haptic feature VR simulator in learning compared with natural tooth. Students made valuable comments towards the representational fidelity features of the VR simulator that the haptic feedback did not provide the same tactile sensation as natural tooth. In this connection, the interaction between students and the simulator could not optimize as expected. This was a valuable information which could be reflected back to the simulator manufacturers for further improvement.

Students suggested that the use of VR simulator training could start earlier at their first-year study at the dental school instead of waiting till their second-year. They also suggested that VR simulation training could also be applied to other courses, such as Direct Restoration and Radiology. These opinions and suggestions were valuable and should be reflected back to the educators for their consideration of training programme and curriculum development. Based on this feedback, another studies evaluating VR is currently conducted to evaluate the effectiveness of using VR on enhancing basic hand skills of first year Bachelor of Dental Surgery (BDS I) students.

This outcome of the current study revealed that majority (57 out of 58 valid returned questionnaires) rated natural tooth as the most effective learning tools in training clinical skills for caries removal. This finding was in agreement with the study from Quinn [29] that students recognized the benefits of VR simulation training; however, they also reported that the VR simulation training could not replace human, subjective interaction. It was perspicuous that the best context would be having natural tooth for practice. Nevertheless, natural tooth is scarce as we need to extract the tooth from patients for training purpose. Alternative training experience would be sought from the VR simulator as this could provide the users extensive training sessions as many times as users want under a risk-free environment without stress. The learning experience was unique and interesting which could motivate users taking the initiation on their own pace and content of learning. Echoing with findings by Hardré et al. [14] in the education context, motivation has been found as a potentially crucial factor affecting the learning effectiveness; consequently, it would influence learners’ performances. The VR haptic enhanced simulator influenced students’ learning behavior to be more engaging and responsible for their own learning pace and practice content in order to enhance their clinical skills. VR haptic enhanced simulator renders its tremendous value in the pre-clinical dental training. Furthermore, this learning tool may improve ‘self-directed learning’ skills of the students.

In this study we faced the limitations of tight dental students’ schedules, and it was not easy to assign extra-sessions for conducting wide-scale assessments. Also, the limited number of relatively expensive haptic simulators did not allow us to conduct the study single time slot. Therefore, it was conducted in serval slots, which required more efforts and supervision. Finally, based in our study findings, it has been highly recommended to implement VR as a supplementary cariology teaching educational tool.

## Conclusion

Virtual reality simulator is a useful learning tool which can benefit undergraduate dental students at their pre-clinical stage; but it cannot totally replace the conventional caries excavation. Despite of the numerous advantages brought from the virtual reality; it could not provide the same tactile sensation compared with conventional caries excavation on natural teeth.

## Data Availability

The datasets used and/or analysed during the current study available from the corresponding author on reasonable request.

## Abbreviations

VR: Virtual Reality
VRLE: Virtual Reality Learning Environment
BDS: Bachelor of Dental Surgery

## Declarations

### Conference presentation

The preliminarily results of this study were presented in The 65th Congress of the European Organization for Caries Research (ORCA), Copenhagen, Denmark (2018).
